# Lysis-independent potentiation of immune checkpoint blockade by oncolytic virus

**DOI:** 10.18632/oncotarget.25614

**Published:** 2018-06-19

**Authors:** Anton Oseledchyk, Jacob M. Ricca, Mathieu Gigoux, Brian Ko, Gil Redelman-Sidi, Tyler Walther, Cailian Liu, Gopa Iyer, Taha Merghoub, Jedd D. Wolchok, Dmitriy Zamarin

**Affiliations:** ^1^ Ludwig Collaborative Laboratory, Memorial Sloan Kettering Cancer Center, New York, NY, USA; ^2^ Swim Across America Laboratory, Memorial Sloan Kettering Cancer Center, New York, NY, USA; ^3^ Department of Medicine, Memorial Sloan Kettering Cancer Center, New York, NY, USA; ^4^ Weill Cornell Medical College, New York, NY, USA; ^5^ Parker Institute for Cancer Immunotherapy, Memorial Sloan Kettering Cancer Center, New York, NY, USA

**Keywords:** NDV, bladder cancer, lysis, immunotherapy, PD-1

## Abstract

Intratumoral therapy with oncolytic viruses is increasingly being explored as a strategy to potentiate an immune response against cancer, but it remains unknown whether such therapy should be restricted to cancers sensitive to virus-mediated lysis. Using Newcastle Disease Virus (NDV) as a model, we explore immunogenic potential of an oncolytic virus in bladder cancer, where existing immunotherapy with PD-1 and PD-L1-targeting antibodies to date has shown suboptimal response rates. Infection of human and mouse bladder cancer cells with NDV resulted in immunogenic cell death, activation of innate immune pathways, and upregulation of MHC and PD-L1 in all tested cell lines, including the cell lines completely resistant to NDV-mediated lysis. In a bilateral flank NDV-lysis-resistant syngeneic murine bladder cancer model, intratumoral therapy with NDV led to an increase of immune infiltration in both treated and distant tumors and a shift from an inhibitory to effector T cell phenotype. Consequently, combination of intratumoral NDV with systemic PD-1 or CTLA-4 blockade led to improved local and abscopal tumor control and overall survival. These findings encourage future clinical trials combining intratumoral NDV therapy with systemic immunomodulatory agents and underscore the rationale for such treatments irrespective of tumor cell sensitivity to NDV-mediated lysis.

## INTRODUCTION

Bladder cancer is the most frequent malignancy of the urinary tract with 79,030 new cases and 16,870 deaths estimated in the United States in 2017 [1]. Most patients (79%) are diagnosed at an early stage with non-muscle-invasive disease [2], and are treated with local transurethral resection of tumor lesions (TURBT), with adjuvant intravesical therapy recommended for high-risk cases [3]. For the last four decades intravesical instillation of the attenuated Bacillus Calmette-Guerin (BCG) has been the gold standard for adjuvant treatment of bladder cancer [4, 5].

In advanced bladder cancer, targeting immune checkpoints such as PD-1 and PD-L1 has demonstrated efficacy [6–10], although benefit from these agents appears to be limited to patients with evidence of pre-existing anti-tumor immune response, exemplified by high numbers of tumor infiltrating lymphocytes (TILs) and PD-L1 expression by tumor cells and tumor-infiltrating immune cells [11, 12]. The development of therapeutic strategies that could improve tumor immune infiltration, potentially rendering them sensitive to therapies with immune checkpoint blockade is needed for the treatement of bladder cancer.

Oncolytic viruses (OV) are an emerging class of therapeutics. Therapeutic effect of oncolytic viruses is mediated by several mechanisms, including direct virus-mediated lysis and activation of tumor-specific immunity [13, 14]. Increasingly, OVs are being evaluated for intralesional/locoregional therapy. In early stage bladder cancer, OVs have been explored with strong signals of efficacy seen in early studies in BCG-refractory patients [15–18]. In addition to local inflammatory response, intratumorally-administered OVs have been demonstrated to incite systemic anti-tumor immunity, as exemplified by intralesional application of talimogene laherparepvec (T-VEC), resulting in its approval for therapy of melanoma by the FDA and EMA [19]. The conversion of the tumor microenvironment by OVs to a more favorable state can improve with the immunomodulatory effects of immune checkpoint blockade, as demonstrated in recent phase I and II trials that showed both, promising response rates as well as acceptable toxicity in combination treatments of OVs with either anti-CTLA4 or anti-PD1 therapy [20–22]. These findings bring up the question of whether locoregional/intratumoral therapy with OVs could be used to potentiate the efficacy of systemic immune checkpoint blockade in bladder cancer.

Replication and lysis are thought to be essential with systemically-administered OVs in order to maximize viral delivery and spread to metastatic lesions. Selection of specific disease types for virotherapy is often based on the preclinical demonstration of the ability of the virus to infect and lyse cancer cells [23]. In the setting of intratumoral therapy, however, the necessity for extensive tumor cell lysis and virus replication is not as clear. For example, we have recently demonstrated that in the setting of pre-existing immunity to an oncolytic virus, reduced virus replication did not attenuate it’s immunotherapeutic efficacy [24].

Given these findings, here we explored whether the extent of lytic effect of OV in bladder cancer cells could be used as a predictor of OV-induced immunogenicity [25]. As a model OV, we use Newcastle Disease Virus (NDV), an avian paramyxovirus with a predilection for a broad range of human tumor cells, robust type I IFN-inducing properties and clinical evidence of anti-tumor activity across multiple tumor types [26–30]. In advanced bladder cancer, anecdotal successes of vaccination with NDV-modified autologous tumor cells have been reported [31]. We demonstrate that infection of murine and a range of human bladder cancer cells with NDV leads to upregulation of MHC proteins, calreticulin, and induction of type I interferon-related genes. Notably, induction of these pathways was seen in all cell lines, independent of the sensitivity of the cell lines to NDV-mediated lysis. *In vivo*, in a bilateral flank syngeneic bladder cancer model resistant to NDV-mediated lysis, intratumoral NDV treatment resulted in increased immune infiltration in treated and distant tumors, rendering them sensitive to therapy with antibodies targeting PD-1 or CTLA-4. These findings highlight that tumor sensitivity to OV-mediated lysis is a poor predictor for immunotherapeutic response and encourage evaluation of intratumoral therapy with NDV in combination with systemic immune checkpoint blockade in clinical trials of advanced bladder cancer.

## RESULTS

### Immunogenic effects of NDV in bladder cancer cells are independent of its lytic potential

**Figure 1:**
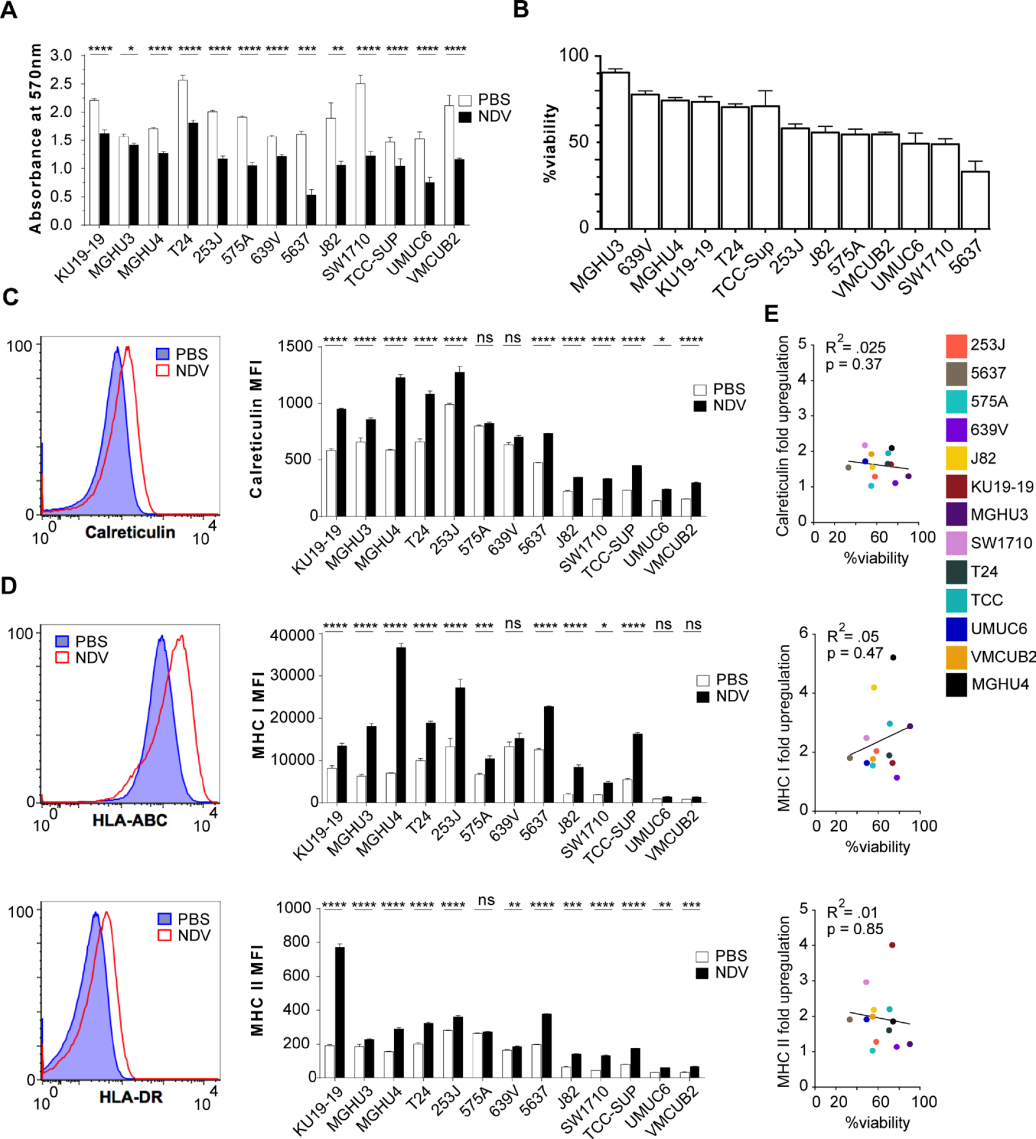
Lytic and immunogenic effects of NDV in human bladder cancer cells Human bladder cancer cell lines were co-cultured with NDV or media for 24 h at a multiplicity of infection (MOI) of 2. (**A**) Viability measured by MTT proliferation assay at 24 hours. (**B**) Percent cell viability calculated from (A). (**C**–**D**) Surface upregulation of calreticulin, MHCI and MHCII at 24 hours quantified by flow cytometry: left: representative histograms using NDV-infected T24 bladder cancer cell line; right: summary bar graphs. (**E**) Correlation of upregulation of calreticulin, MHC I, and MHC II with % viability in NDV-treated human bladder cancer cell lines. ^*^p < 0.05, ^**^p < 0.01, ^***^p < 0.001, ^****^p < 0.0001, ns: non-significant. MFI: median fluorescence intensity. Data represent one of 2 independent experiments with 3 replicates per group.

To examine the sensitivity of bladder cancer cells to NDV-mediated lysis, 13 different human bladder cancer cell lines were treated for 24 h with NDV at a multiplicity of infection (MOI) of 2 and assessed for viability using MTT proliferation assay (Figure 1A, 1B). While there was reduction in viability in every bladder cancer cell line tested, oncolytic sensitivity range was quite variable across the cell lines. To examine whether NDV infection upregulates markers that could potentially lead to improved immune recognition, markers of antigen presentation and immunogenic cell death were assessed. Eleven of 13 cell lines showed a significant increase in surface calreticulin expression, a known marker of immunogenic cell death (Figure 1C). In addition, 12 of 13 cancer cell lines exhibited upregulation of MHC class II, and 10 of 13 exhibited upregulation of MHC class I, highlighting the potential for improved antigen presentation on cancer cells by NDV (Figure 1D). Upregulation of MHC I, MHC II, or calreticulin exhibited no correlation with sensitivity to NDV-mediated lysis, suggesting that even in the setting of poor lytic response, infection with NDV results in modification of surface protein profile to a more favorable one for immune recognition (Figure 1E). Notably, in bladder cells infected with NDV expressing GFP (NDV-GFP), upregulation of MHC I and MHC II was seen in both virus-infected (GFP+) cells and in non-infected cells, marked by lack of GFP expression (Supplementary Figure 1).

**Figure 2:**
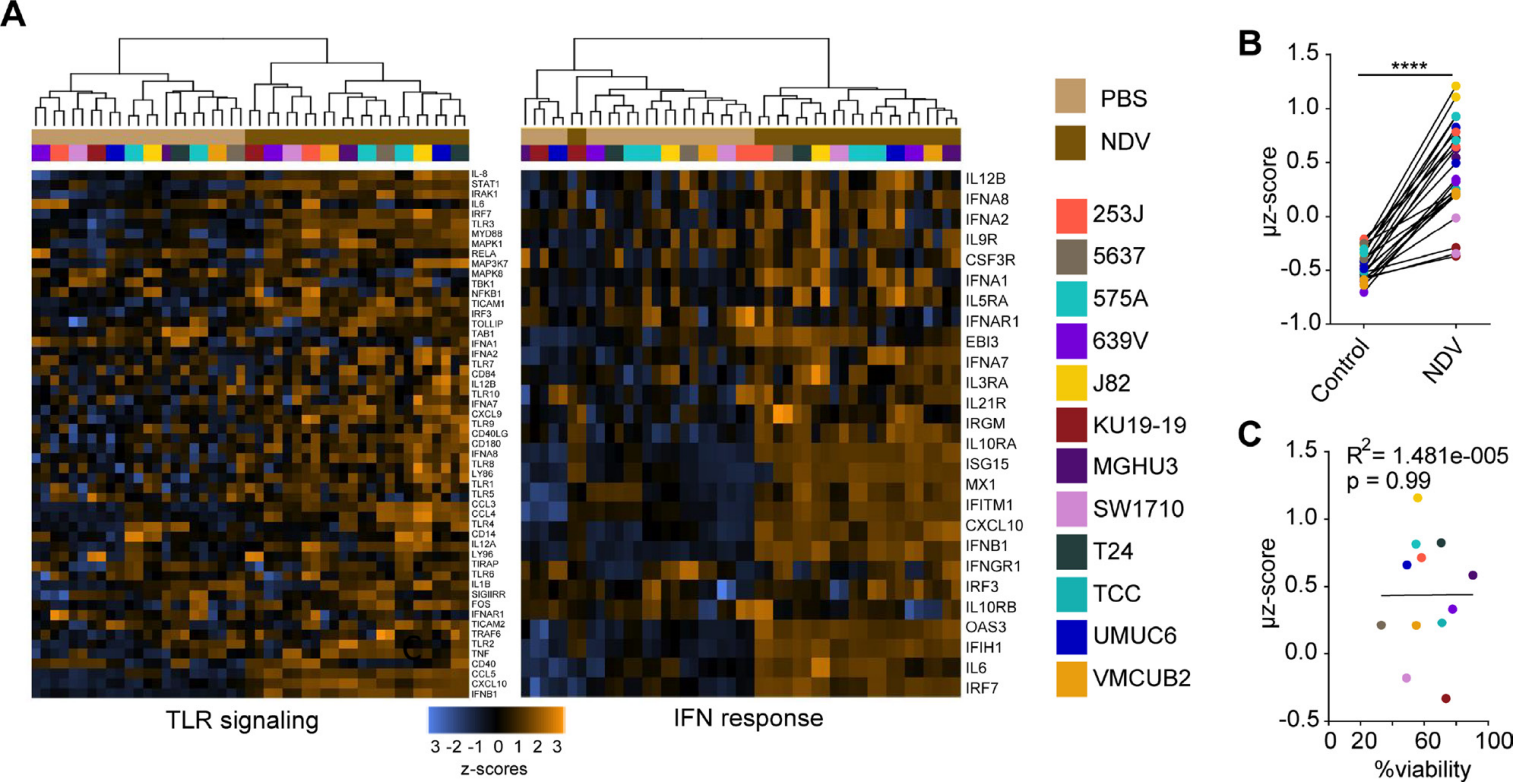
NDV induces type 1 interferon response in human bladder cancer cells Gene expression analysis was performed using the Innate Immunity Panel profiling kit on the Nanostring platform. (**A**) Upregulation of gene sets related to TLR signaling and type I interferon (IFN) response. (**B**) Type I IFN markers were used to calculate an activation signature µ z-score. (**C**) Correlation of µ z-score vs. % viability. ^****^p < 0.0001. Data represent a single experiment with 2 replicates per cell line.

Given the uniform upregulation of MHC I and II irrespective of lysis and direct infection, we reasoned that these alterations could be related to activation of innate immune response. To examine the innate immune pathways activated in response to NDV on a broad transcriptional level, a comparison of gene expression of naïve and NDV-infected human bladder cancer lines was performed on the Nanostring platform using the Innate Immunity Panel profiling kit. Across all tested cells, there was a strong upregulation of a range of genes known to promote innate immune recognition, including components of TLR signaling pathways and type I IFN response-related genes (Figure 2A). Using the Nanostring type I IFN signature gene set, a mean z score (μz) was generated for each cell line, demonstrating upregulation of the signature in all cell lines tested (Figure 2B). Similar to the examined surface markers, there was no association between the type I IFN signature and the degree of NDV-mediated lysis (Figure 2C). In addition to upregulation of type I IFN and antigen presentation markers, we observed upregulation of a range of chemokines and cytokines known to mediate recruitment and proliferation of adaptive immune cells (Supplementary Figure 2).

**Figure 3:**
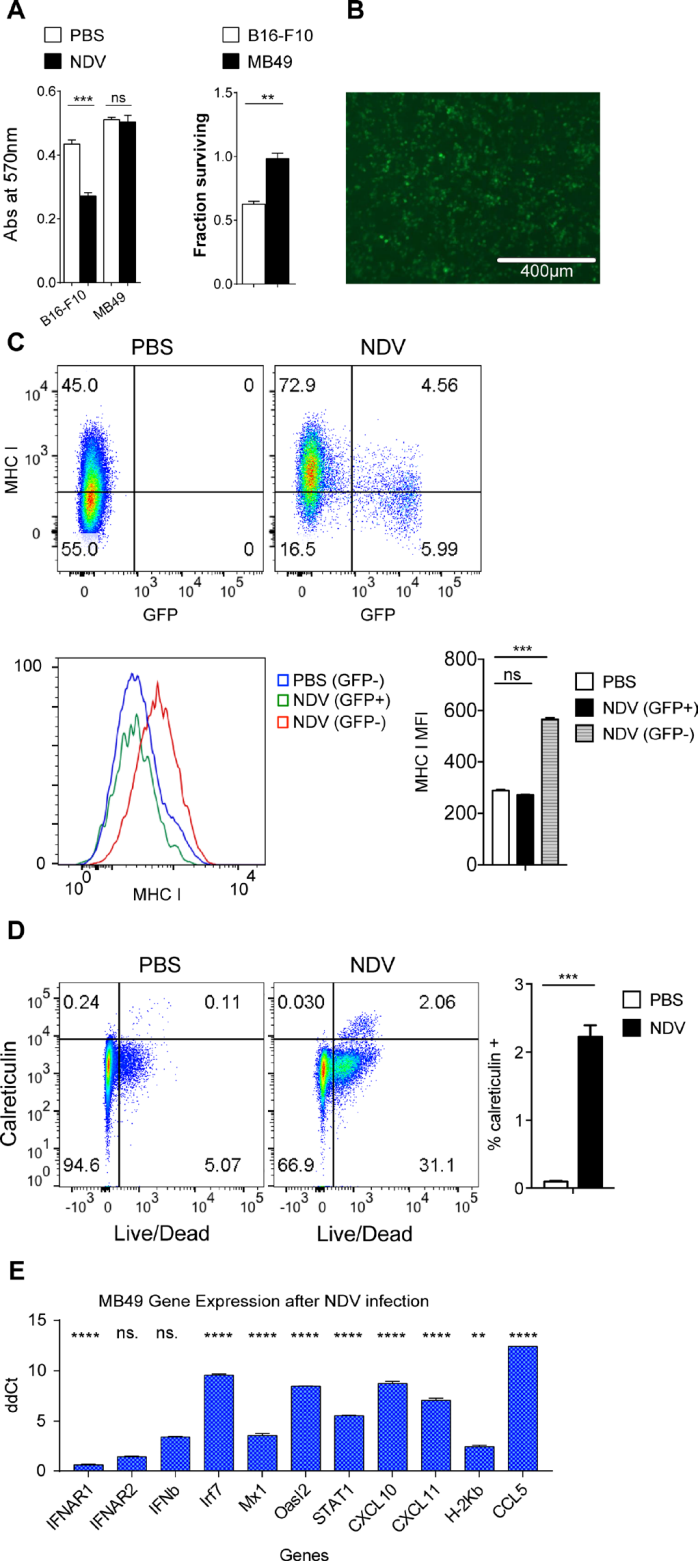
NDV infection of murine bladder cancer line exhibits similar effects as in human bladder cancer cell lines MB49 and B16-F10 cells were infected with NDV at a MOI of 2. (**A**) Viability measured by MTT proliferation assay at 24 hours; left: absolute absorbance at 570 nm, right: viability calculated from absorbance. (**B**) GFP expression at 24 hours in MB49 cells after infection with NDV-GFP at MOI of 2. (**C**) Expression of MHC I on murine bladder cancer cell line MB49 in response to NDV-GFP infection for 24 h. Top: Representative flow cytometry plots demonstrating MHC I expression in relation to GFP. Bottom: MFI of MHC I in PBS-treated cells and in the infected (GFP+) and non-infected (GFP−) cells treated with NDV. (**D**) Upregulation of calreticulin in MB49 cells at 24 hours. Left: representative flow cytometry plots; right: summary bar graph. (**E**) Fluidigm gene expression analyses of NDV infected MB49 cells compared to naïve MB49 cells focusing on selected type I interferon genes. ^**^p < 0.01, ^****^p < 0.0001, ns: non-significant. MFI: median fluorescence intensity. Data represent one of 2 independent experiments with 3 replicates per group.

These findings thus suggested that immunogenic effects of NDV may be independent of its lytic effect. Given the known key role of the type I IFN pathway in antigen presentation by CD8+ dendritic cells (DCs) [32–34], as well as association of chemokines with tumor T cell infiltration [35], we proceeded to evaluate whether similar response patterns could be recapitulated in murine bladder cancer MB49, which is resistant to NDV-mediated lysis. Infection of MB49 cells with NDV expressing GFP (NDV-GFP) revealed evidence of infection, but complete lack of lytic effect, in comparison to the melanoma cell line B16-F10, where the virus previously demonstrated *in vivo* efficacy (Figure 3A–3B). Despite the poor lytic effect, there was robust upregulation of surface MHC class I expression (Figure 3C) and an increase in surface calreticulin-positive cells (Figure 3D). Similarly to human cell lines, upregulation of MHC I was primarily limited to non-infected cells, marked by lack of GFP expression (Figure 3C), suggesting that it was likely driven by a paracrine effect of innate immune response activated in the infected cells. Indeed, there was a marked upregulation of the majority of the tested type I IFN-related genes (Figure 3E). Type II IFN-related genes like IFNGR1, IFNGR2, FA Pa, IDO, IFNγ, Tbx21 and CXCL9 did not show significant increase or remained undetectable by qPCR in both infected and naïve MB49 cells (data not shown).

### Intratumoral NDV therapy results in local and distant treatment effect and expansion of cytotoxic and effector lymphocytes

**Figure 4:**
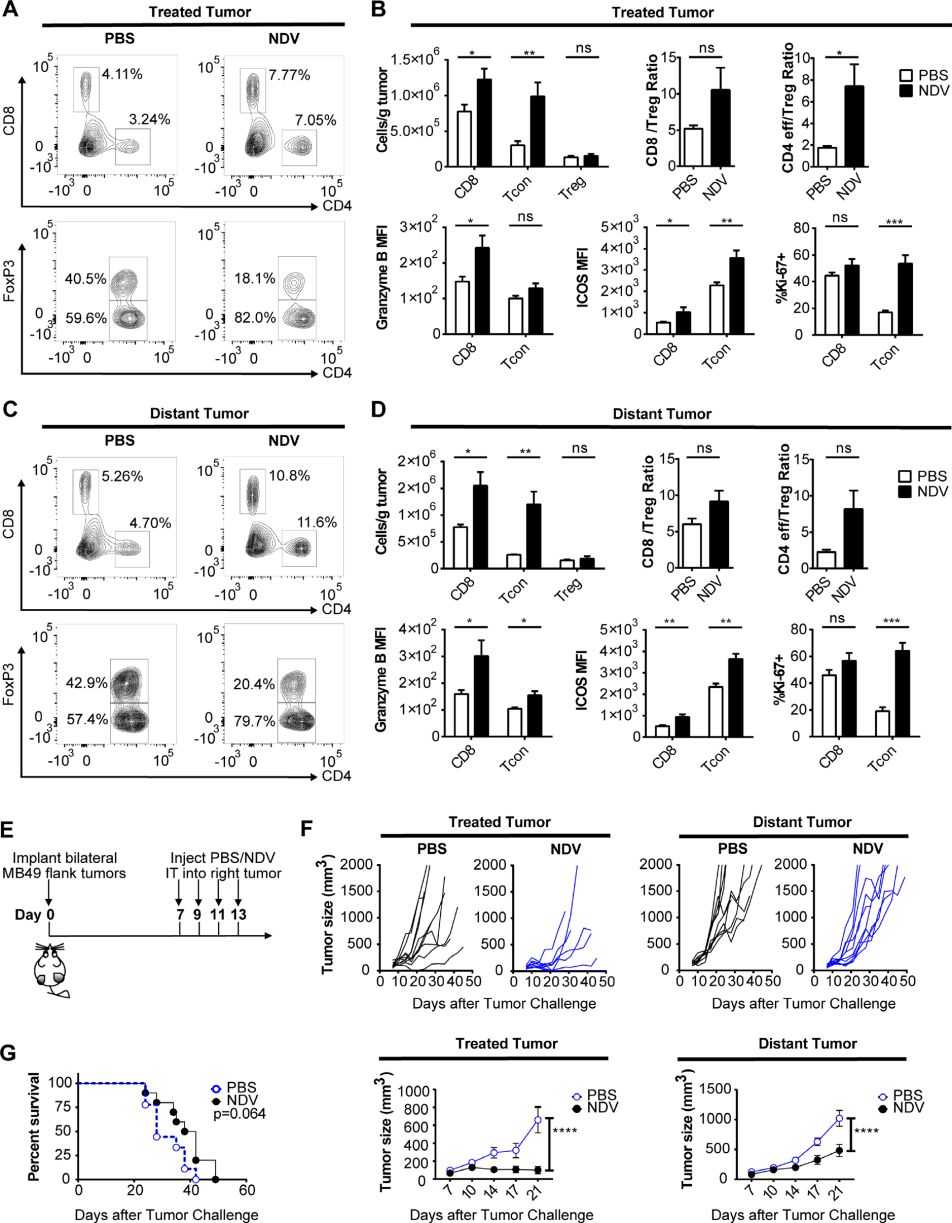
NDV induces increased immune infiltration and delay of tumor growth in treated and distant tumors Animals bearing bilateral flank MB49 bladder tumors were treated with 3 injections of NDV administered every other day into right tumor. (**A**–**D**) The tumors were collected 3 days after last treatment and analyzed by flow cytometry. (A) Representative flow cytometry plots showing proportion of CD4+ and CD8+ cells (% of all live single cells) (top) and proportion of Foxp3− and FoxP3+ CD4 cells (% of all CD4) (bottom) in tumors treated with PBS (left) or NDV (right). (B) T cell infiltration in the treated tumors and expression of activation (ICOS), lytic (GrB), and proliferation (Ki-67) markers by the CD8 and Tcon lymphocytes from treated tumors. (C) Representative flow cytometry plots showing proportion of CD4+ and CD8+ cells (%of all live single cells) (top) and proportion of Foxp3− and FoxP3+ CD4 cells (%of all CD4) (bottom) in distant tumors of mice treated with PBS (left) or NDV (right). (D) T cell infiltration in the distant tumors and expression of activation (ICOS), lytic (Granzyme B), and proliferation (Ki-67) markers by the CD8 and Tcon lymphocytes from distant tumors. (**E**–**G**) Animals bearing bilateral flank MB49 bladder tumors were treated with 4 injections of NDV administered every other day into right tumor. (E) Treatment schema. (F) Growth of treated and distant tumors and mean tumor growth curves (± SEM) compared at day 21 post treatment. (G) Overall survival. Data demonstrate representative results from two independent experiments with 5–10 animals per group. ^*^p < 0.05, ^**^p < 0.01, ^***^p < 0.001, ^****^p < 0.0001, ns: non-significant. MFI: median fluorescence intensity; Tcon: conventional T cell (CD4+FoxP3−); Treg: regulatory T cell (CD4+FoxP3+). (A–D) Data represent one of 2 independent experiments with 5 mice per group. (E–G) Data represent one of 2 independent experiments with 10 mice per group.

These findings suggest that even in the setting of poor oncolysis of cancer cells, the immunostimulatory activity of NDV has a potential to promote tumor immune recognition. To explore whether this would be reflected in anti-tumor activity *in vivo*, we evaluated the effects of intratumoral NDV therapy in a bilateral flank MB49 bladder cancer model, with the virus administered to a single-flank tumor. Such a model allowed us to measure both direct immune effects of NDV on the infected tumor, and indirect or abscopal effects on the distant tumors. Flow cytometry analysis of both treated and distant tumors demonstrated a marked increase in the infiltrating lymphocytes (Figure 4A–4D). Notably, there was a significant increase in the number of infiltrating CD8 and conventional CD4+ Foxp3− T cells (Tcon) with only a minor increase in CD4+FoxP3+ regulatory T cells (Treg) (Figure 4A–4D), resulting in increasing Tcon to Treg ratios (Figure 4B, 4D). Additionally, both CD8+ and Tcon showed increased expression of activation (ICOS), lytic (GrB), and proliferation (Ki-67) markers in both treated and distant tumors (Figure 4B, 4D).

To assess the efficacy of single-agent intratumoral NDV, animals bearing bilateral flank MB49 tumors were treated intratumorally into the right flank tumor for a total of 4 treatments (Figure 4E). This treatment significantly delayed tumor growth and induced complete rejection of some treated tumors (Figure 4F). Since the MB49 cell line is poorly susceptible to NDV-mediated lysis (Figure 3A), we speculate that tumor control in this setting was primarily immune mediated. In addition there was a delayed growth of the distant tumors (Figure 4F), albeit to a more modest extent, which resulted in eventual outgrowth of all tumors with no statistically-significant improvement in overall survival (Figure 4G). This suggests that while NDV can establish good locoregional tumor control and regression of the injected tumors, the abscopal immune effect is insufficient for long-term control of distant tumors, despite favorable inflammatory changes. This prompted us to examine whether immune checkpoint pathways controlling T cell activation play a role in preventing complete tumor rejection.

### Combination therapy of intratumoral NDV and systemic immune checkpoint blockade induces durable responses to treatment

**Figure 5:**
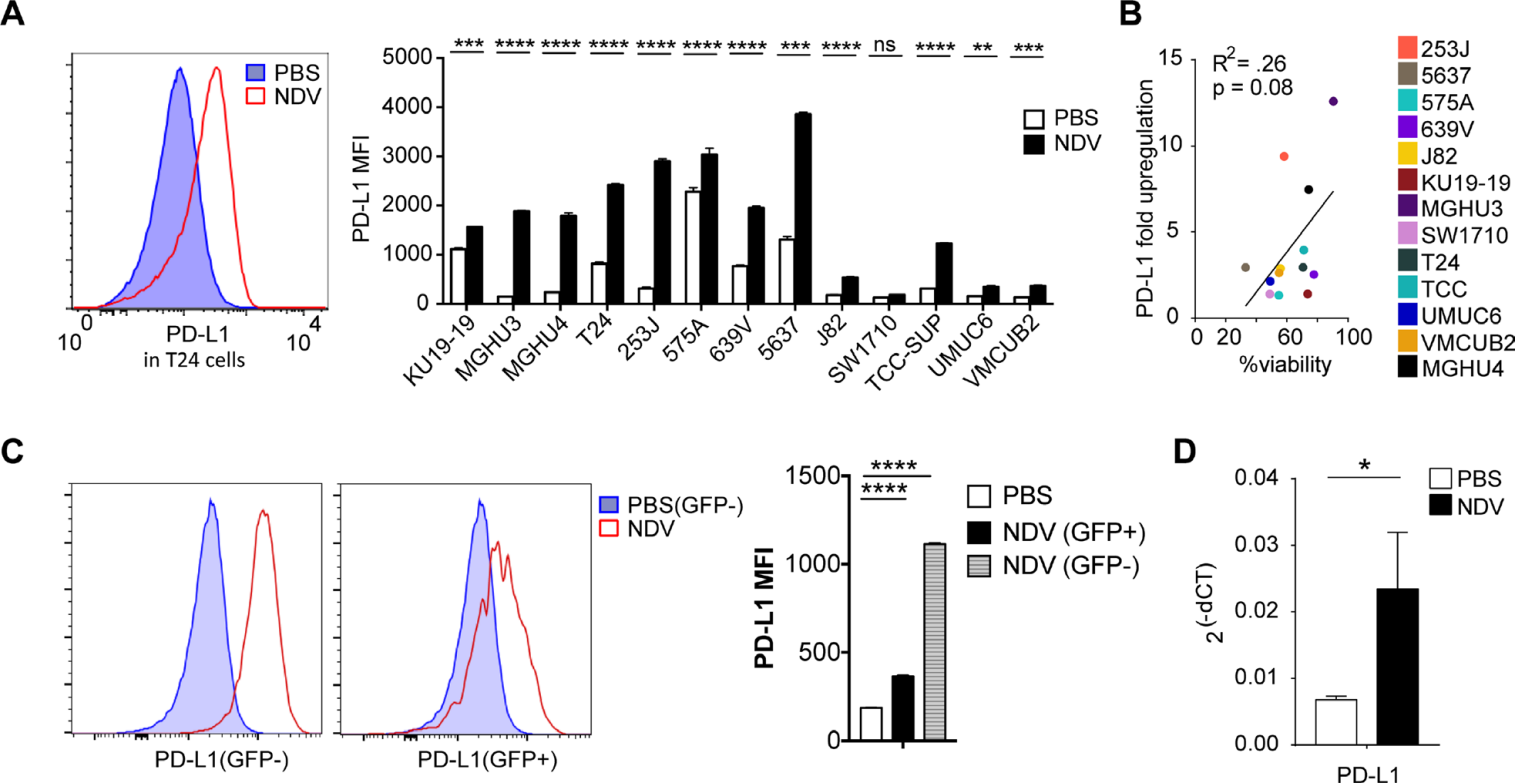
NDV treatment induces PD-L1 overexpression in bladder cancer lines Human bladder cancer cell lines were co-cultured with NDV or media for 24 h at a MOI of 2. (**A**) Upregulation of PD-L1 in human bladder cell lines. left: representative histogram of PD-L1 expression in T24 cell line; right: summary bar graphs. (**B**) Correlation of upregulation of PD-L1 vs. % viability in NDV-treated human bladder cancer cell lines. (**C**) PD-L1 upregulation in the MB49 murine bladder cancer cells treated with NDV-GFP. Left: representative histograms demonstrating PD-L1 MFI in PBS-treated and NDV-treated infected (GFP+) and non-infected (GFP−) cells. Right: summary bar graphs. (**D**) PD-L1 upregulation quantified by Fluidigm in NDV-treated tumors. ^*^p < 0.05, ^****^p < 0.0001. Data represent one of 2 independent experiments with 3 replicates per group. Mean ± SEM are shown.

**Figure 6:**
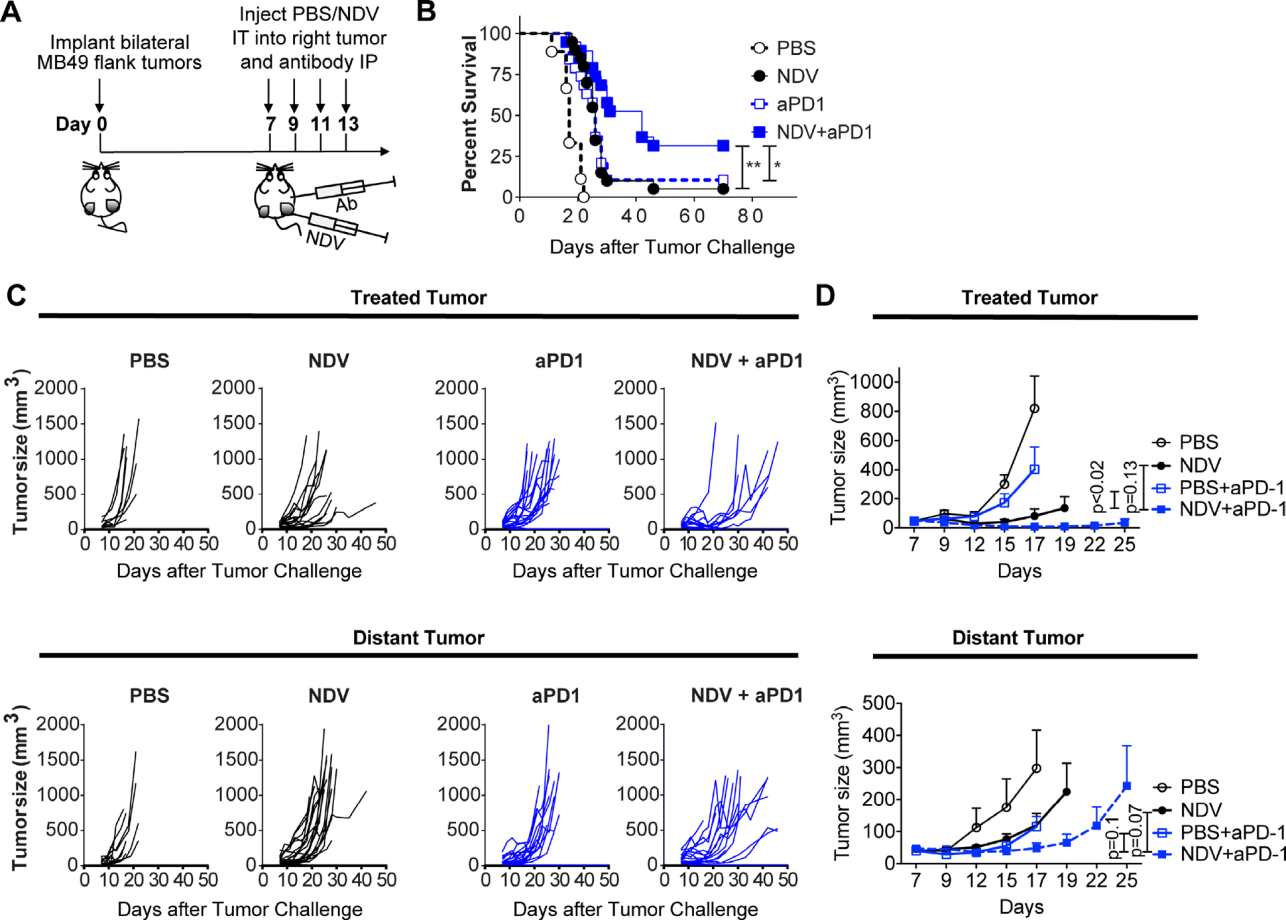
PD-1 blockade potentiates the regression of NDV-treated and distant tumors Animals bearing bilateral flank MB49 bladder tumors were treated with 4 injections of NDV administered every other day into right tumor combined with concomitant intraperitoneal injection of aPD-1 antibodies or PBS. (**A**) Treatment schema. (**B**) Overall Survival. (**C**) Growth of individual treated and distant tumors. (**D**) Mean tumor volumes. Tumor growth curves stop at the time of first animal death in each group. Statistical comparisons were performed at the time of first animal death. (B–C) data pooled from two independent experiments with 5–10 mice per group. (D) Representative experiment with 5–10 mice per group. ^*^p < 0.05, ^**^p < 0.01. Mean ± SEM are shown.

Phenotypic analysis of bladder cancer cell lines in response to NDV infection revealed marked surface upregulation of PD-L1 in 12 of the 13 human cell lines (Figure 5A) without significant correlation between viability and PD-L1 upregulation (Figure 5B). Similarly, we observed upregulation of PD-L1 in the MB49 cell line, which was observed in both virus-infected (GFP+) and non-infected cells (GFP−) (Figure 5C). *In vivo*, we observed upregulation of PD-L1 gene expression in the treated MB49 tumors in response to NDV (Figure 5D). These findings created a rationale for evaluation of combination therapy of intratumoral NDV with systemic PD-1 blockade. Animals were treated with 4 concomitant intratumoral NDV injections and intraperitoneal aPD1 antibody injections in a bilateral MB49 tumor model (Figure 6A). While NDV or aPD1 alone had only a modest impact on tumor growth and survival, combination therapy resulted in significantly delayed tumor growth of treated and distant tumors with a complete rejection of bilateral tumors in 32% of the mice (Figure 6B–6D).

**Figure 7:**
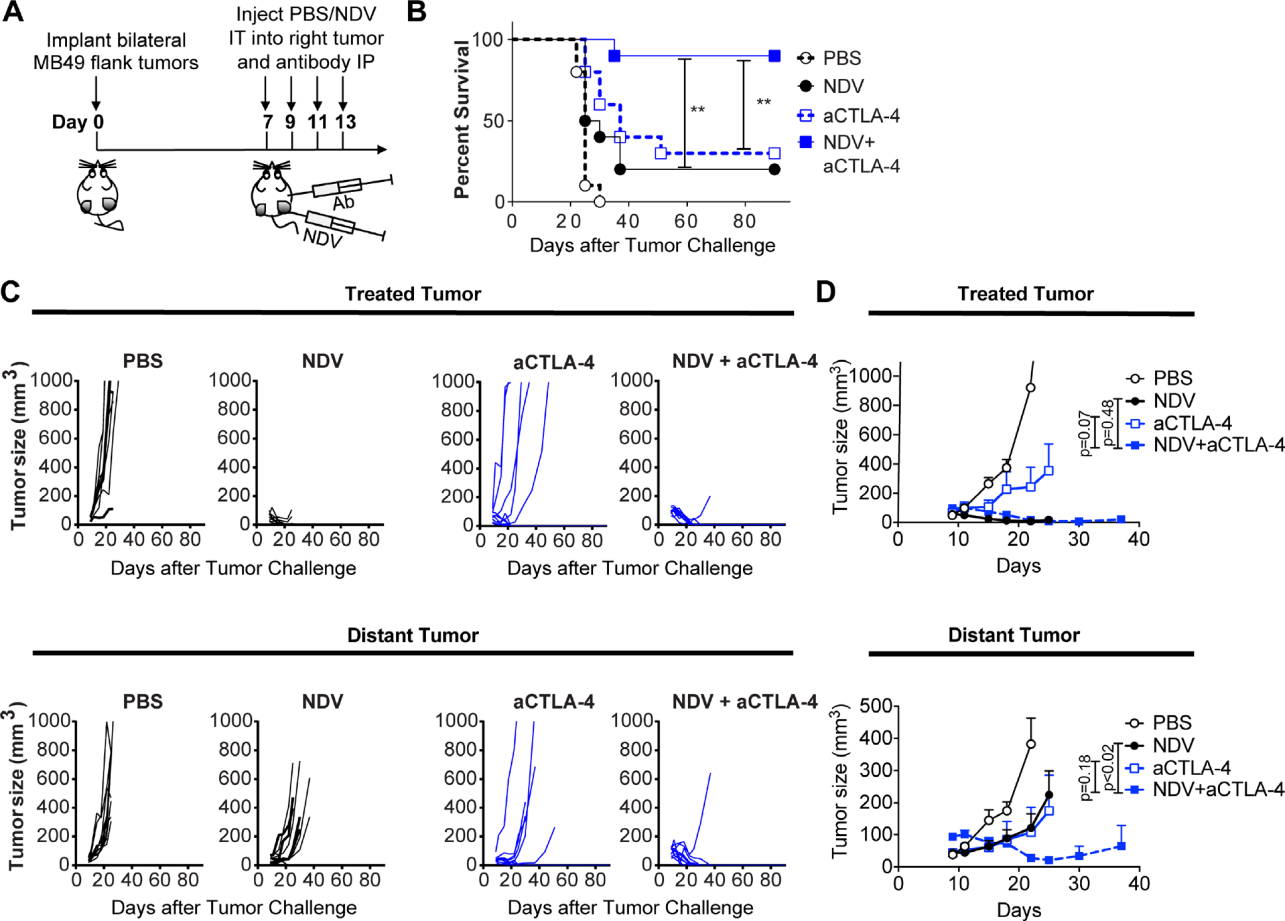
CTLA-4 blockade potentiates the regression of NDV-treated and distant tumors Animals bearing bilateral flank MB49 bladder tumors were treated with 4 injections of NDV administered every other day into right tumor combined with concomitant intraperitoneal injection of aCTLA-4 antibodies or PBS. (**A**) Treatment schema. (**B**) Overall Survival. (**C**) Growth of individual treated and distant tumors. (**D**) Mean tumor volumes. Tumor growth curves stop at the time of first animal death in each group. Statistical comparisons were performed at the time of first animal death in NDV and CTLA-4 group (day 25). Error bars represent standard error of the mean. Data demonstrate representative results from one of two independent experiments with 5–10 animals per group). ^**^p < 0.01.

To expand on these findings, we explored whether CTLA-4 blockade in combination with NDV could also be employed in this setting, given the activity of ipilimumab in urothelial cancer patients [36]. Given the aggressive nature of the MB49 model and a relatively modest effect of single-agent CTLA-4 blockade seen in humans, a lower tumor challenge was used compared to the PD-1 combination experiments. In this setting, while NDV or CTLA-4 blockade had modest activity as single agents, the combination treatment resulted in long-term survival in 90% of animals (Figure 7A, 7B) by achieving a complete rejection of the majority of treated and distant tumors (Figure 7C, 7D). While these results cannot be directly compared to the NDV combination with PD-1 blockade due to experimental differences, they nevertheless indicate that CTLA-4 blockade is an attractive combination partner for intratumoral NDV therapy. Overall, these findings suggest that the combination of *in situ* vaccination with oncolytic viruses has a potential to improve responses to immune checkpoint blockade in bladder cancer.

## DISCUSSION

Despite the promise of immune checkpoint blockade in bladder cancer, tumor responses remain limited to the minority of patients. Studies have demonstrated that expression of PD-L1 in tumor cells and tumor-infiltrating immune cells in bladder cancer can select for patients that are more likely to benefit from such treatments [12, 37]. Consequently, strategies that can increase intratumoral T cell infiltration and PD-L1 upregulation could theoretically enhance the efficacy of immune checkpoint blockade [38].

*In situ* vaccination strategies employ approaches using the patient’s own tumor as a vaccine through immunogenic cell death, characterized by release of tumor antigens and provision of appropriate immune signals to promote activation of tumor-specific immunity. These approaches span a multitude of approved and experimental techniques, and rely on abscopal effects to induce tumor shrinkage at distant sites. *In situ* vaccination using ablative strategies, such as radiation and cryoablation, and intratumoral injection of a variety of immunomodulatory agents, such as TLR and STING agonists, have demonstrated activity in preclinical models and have recently entered the clinical arena, alone and in combination with immunomodulatory antibody therapy [39–43].

Oncolytic viruses may exhibit certain advantages over other types of *in situ* vaccination [22]. In addition to lysis of cancer cells and release of danger signals and tumor antigens, OVs can infect and activate antigen-presenting cells directly. OVs may also maintain better persistence within tumors due to their replicative ability. Finally, OVs can be genetically modified to deliver additional immunostimulatory ligands or antigens directly into the tumor. One of the limitations of OVs is their poor delivery to the tumor sites with systemic administration due to virus sequestration, neutralizing antibodies, and lack of extravasation. As such, for systemic therapy, administration of a replicating OV vector maximizes the chances of successful tumor infection by allowing multicycle virus replication and spread through the tumor. In the setting of intratumoral therapy, it remains unclear, however, whether sensitivity of cancer cells to OV-mediated lysis is required for the OV-induced immune effects, as even low-productive infection carries a potential to induce anti-tumor immune response. This is exemplified by studies demonstrating that heat-inactivated or non-replicating viruses still carry ability to mediate tumor control and regression [44, 45]. In the current study we sought to determine whether sensitivity to OV-mediated lysis could predict the degree of immune activation in the infected cancer cells and to evaluate the efficacy of OV in a murine model resistant to OV-mediated lysis. We specifically chose to focus on bladder cancer, given the history of success of both locoregional (BCG) and systemic (anti-PD-1, anti-PD-L1) immunotherapeutic approaches and the need for more effective therapeutic combinations in this disease.

NDV is a negative strand RNA virus, which has previously demonstrated to be safe and had evidence of clinical activity including partial and complete responses with systemic administration in patients with various tumor types [46]. In addition, the virus has been explored as a vaccine agent in bladder cancer, using vaccination with autologous virus-modified cancer cell lysates [31]. Here, we explored the effects of NDV in human and murine bladder cancer cell lines. We demonstrate that the lytic effect of NDV is quite variable, with several cell lines being very resitant to NDV-mediated lysis. Irrespective of the degree of lysis, however, the majority of the cell lines demonstrated upregulation of surface calreticulin, and induction of MHC class I and II. There was no correlation between these effects and the degree of tumor cell lysis, which suggests that induction of the immunogenic markers is mediated by the innate immune pathways activated by NDV. In concordance with this, we observed a strong upregulation of type I IFN-related genes, which again did not correlate with the degree of lysis. Interestingly, the most robust upregulation of MHC I was seen in the cells not directly affected by virus infection (Figure 3C), provoking a question of whether extensive virus-mediated lysis could actually be detrimental to antigen presentation.

Consistent with the *in vitro* findings, intratumoral therapy with NDV *in vivo* led to favorable changes in the tumor microenvironment, with increase in tumor-infiltrating lymphocytes in both virus-injected and distant tumors. In line with our previous studies, such therapy was insufficient to induce complete rejection of the virus-injected or distant tumors, likely secondary to negative feedback immune mechanisms activated in response to NDV infection and immune infiltration. In B16-F10 melanoma model, we have recently demonstrated that therapy with NDV can lead to upregulation of PD-L1, an effect that is mediated both by type I IFN early in response to direct virus infection, and by response to increased TILs late in treatment [47]. Here, we similarly observe surface upregulation of PD-L1 in human and mouse bladder cell lines. A uniform increase in PD-L1 MFI in cell lines, as well as upregulation of PD-L1 in cells that lack direct evidence of infection suggest that paracrine effect of activation of innate immune pathways could be the primary mechanism driving this increase [47].

An increase in TILs and upregulation of PD-L1 generate a strong rationale for combination of intratumoral NDV with systemic immune checkpoint blockade using PD-1 or CTLA-4. Indeed, we demonstrate that such combinations were able to achieve regression of both virus-injected and distant tumors. Several recent studies have explored different OVs in combination with systemic PD-1/PD-L1 blockade in different pre-clinical tumor models. The majority of the studies have demonstrated additive or synergistic effects between the two treatment modalities in the virus-injected tumors; however, very few studies have been conducted to determine the utility of such therapies against distant, non-virus-injected tumors [47–55]. Recently, two studies using oncolytic T-Vec in metastatic melanoma patients have demonstrated promising response rates when administered in combination with CTLA-4 or PD-1 blockade [20, 21]. Based on our findings, there is a strong rationale for exploration of similar strategies in bladder cancer, whereby an OV could be administered intralesionally in combination with systemic immune checkpoint blockade. One may also envision moving such strategies into an earlier disease setting, to prevent cystectomy and decrease the risk of development of advanced disease. Our study suggests that sensitivity of cancer cells to NDV-mediated lysis is not a prerequisite for an efficacy of such an approach, although virus entry and possibly limited replication is likely still required to achieve adequate activation of innate immune response. Thus, it is unknown whether these findings could be generalized to the setting of an absolute resistance to OV infection, for example due to lack of cellular receptor for the virus. Given the ubiquitous expression of NDV receptor (sialic acid) on all cell types, the latter is unlikely to be a limiting factor for NDV.

In summary, these findings demonstrate that sensitivity of tumor cells to NDV do not predict its efficacy *in vivo* and provide a rationale for NDV-based *in situ* vaccination strategy in combination with systemic immune checkpoint blockade in patients with bladder cancer.

## MATERIALS AND METHODS

### Mice

C57BL/6J mice were purchased from Jackson Laboratory. All mice were maintained in microisolator cages and treated in accordance with the NIH and American Association of Laboratory Animal Care regulations. All mouse procedures and experiments for this study were approved by the Memorial Sloan Kettering Cancer Center Institutional Animal Care and Use Committee.

### Cell lines

The murine bladder cancer cell line for melanoma (MB49, originally generated by Dr. Ian Summerhayes at Imperial Cancer Research Fund, UK) as well as the human bladder cancer cell lines (originally provided by Dr. Dan Theodorescu) were cultured in complete RPMI medium (10% fetal calf serum and penicillin with streptomycin). Cell lines were tested and found to be negative for mycoplasma contamination. The cell lines have not been re-authenticated since their receipt from the original sources. A549 cell line was obtained from ATCC.

### Antibodies

Therapeutic anti-PD-1 (clone RMP1-14) and anti-CTLA4 (9H10) monoclonal antibodies were produced by BioXcell. Antibodies used for flow cytometry were purchased from the following sources (dilutions are indicated in parentheses): eBioscience (CD45.2 Alexa Fluor 700, cat: 56-0454 (1:200), CD3 PE-Cy7, cat: 25-0031 (1:200), CD4 ef450, cat: 48-0041 (1:200), CD8 PerCP-efluor710, cat: 46-0083 (1:200), CD11b APC-efluor 780, cat: 47-0112 (1:600), ICOS PE, cat: 12-5985 (1:200), PD-L1 PE Cy7, cat: 25-5982-82 (1:200), FoxP3 Alexa Fluor 700, cat: 56-5773 (1:100), FoxP3 APC, cat: 17-5773 (1:200), PD-1 PE-Cy7, cat: 25-9985 (1:200)), Invitrogen (Granzyme B PE-Texas Red, cat: GRB17 (1:125)) and BD Pharmingen (Ki67-Alexa Fluor 488, cat: 561165 (1:50)).

### Viruses

Recombinant lentogenic NDV LaSota strain was used for all experiments. Generation of recombinant viruses was described previously [56]. Virus titers were determined by serial dilution and immunofluorescence in A549 cells.

### *In vitro* infection experiments

Indicated cell lines were cultured to 80% confluency and were infected in 6-well dishes at a multiplicity of infection (MOI) of 2, which is the number of viruses per cell, in a total volume of 3ml of RPMI medium supplemented with 10% fetal calf serum and penicillin with streptomycin. Twenty-four hours later, the cells were harvested by scraping and processed for PD-L1, MHC I, MHCII and calreticulin surface labeling and quantification by flow cytometry or gene expression analysis. For viability, the cells were assessed using MTT proliferation assay (Promega) at 24 h after infection according to the manufacturer’s instructions. Detergent reagent was added, and two hours later the plate was measured for absorbance at 570 nm wavelength in SpectraMAX 340PC (Molecular Devices). Viability was calculated from absorbance of MTT assay with the following formula: (absorbance in infected cells– absorbance background)/(absorbance in control cells–absorbance background) × 100.

### Tumor implantation and survival experiments

Bilateral flank tumor models were established using cell dose and schedule that would allowe for evaluation of both local and abscopal effects. Tumors were established by injection of 2 × 10^5^ MB49 cells in both flanks intradermally (i.d.) on day 0. On days 7, 9, 11, and 13 the mice were treated with intratumoral injections of 1 × 10^7^ pfu of NDV in PBS in a total volume of 100 μl. Concurrently, where indicated, on days 7, 9, 11, and 13 the mice received intraperitoneal (i.p.) injections of anti-PD-1 antibody (250 μg). In the experiments involving combination of NDV with anti-CTLA-4 antibody, tumors were established by injection of 1 × 10^5^ MB49 cells in both flanks intradermally (i.d.) on day 0. On days 7, 9, 11, and 13 the mice were treated with intratumoral injections of 1 × 10^7^ pfu of NDV in PBS in a total volume of 100 μl. Concurrently, where indicated, on days 7, 9, 11, and 13 the mice received intraperitoneal (i.p.) injections of anti- anti-CTLA4 antibody (100 μg). Control groups intraperitoneal and intratumoral injections of PBS. The animals were euthanized for signs of distress or when the total tumor volume (largest diameter × smallest diameter × depth/2) reached 1000 mm^3^.

### Isolation of tumor-infiltrating lymphocytes

MB49 tumors were implanted by injection of 2 × 10^5^ MB49 cells in both flanks i.d on day 0. On days 7, 9, and 11 the mice were treated with intratumoral injections of 1 × 10^7^ pfu of NDV or intratumoral injection of PBS. To ensure sufficient tumor volume for processing and TIL characterization, only 3 doses of intratumoral NDV injections were administered. On day 14, mice were euthanized and tumors and tumor-draining lymph nodes were removed using forceps and surgical scissors and weighed. Time for TIL assessment was established in previous studies [26, 47, 54] and allowed to evaluate all of the control animals before euthanasia for large tumor burden and all treated animals before complete tumor clearance. Tumors were minced with scissors prior to incubation with 1.67 Wünsch U/mL Liberase and 0.2 mg/mL DNase for 30 minutes at 37° C. Tumors were then homogenized by repeated pipetting and filtered through a 70-μm nylon filter. Cell suspensions were washed once with complete RPMI and processed for flow cytometry. Cells from tumor draining lymph nodes were isolated by grinding the lymph nodes through a 40 μm nylon filter.

### Flow cytometry

Cells isolated from tumors or tumor-draining lymph nodes were processed for surface labeling. Fixable viability dye eFluor506 (eBioscience) was used to distinguish the live cells. Cells were further permeabilized using FoxP3 fixation and permeabilization kit (eBioscience) and stained for Ki-67, FoxP3, Granzyme B. Data was acquired using the LSRII Flow cytometer (BD Biosciences) and analyzed using FlowJo software (Treestar).

### NanoString gene expression analyses

Infected human bladder cancer cell lines and non-infected controls were scraped from plate and placed in TRIzol reagent (Invitrogen), and homogenized. The samples were flash-frozen in dry ice and ethanol and stored at −80° C. RNA was later purified from TRIzol using the Direct-zol RNA MiniPrep kit (Zymo Research). Isolated RNA was hybridized with the NanoString Innate Immunity Profiling human panel codeset (N2_HS_IN_IMM_V1.0) and quantified using the nCounter Digital Analyzer at the MSKCC Genomics Core Facility. Data were processed with nSolver Analysis Software (version 3.0.22), using the Advanced Analysis module.

### Quantitative real-time PCR

MB 49 cells were homogenized immediately after detachment and RNA was extracted using the RNeasy Mini Kit (Qiagen) according to manufacturer instruction. The samples were stored at −80° C until reverse transcription PCR was performed with High Capacity cDNA Reverse Transcription Kit (Applied Biosystems). No preamplification of cDNA products was performed. For real-time PCR, VeriQuest Probe qRT-PCR Master Mix (2X) (Affymetrix) and TaqMan^®^ Gene Expression Assays (Thermo Fisher Scientific) for each gene investigated, as well Actb and Gapdh as housekeeping genes, were purchased. Real-time PCR was performed on the Fluidigm Biomark HD platform on a 48.48 IFC chip with provided reagents and according to manufacturer’s instructions (User Guide: PN 68000089 H1). Real-time PCR results were analyzed in Real-Time PCR Analysis v4.3.1 software (Fluidigm). Gene expression levels were normalized to *Gapdh* (Δ*C*_t_ = *C*_t_ *gene* – *C*_t_ *Gapdh*) and reported as relative mRNA expression (2^−(Δ*C*t)^).

### Statistics

Data were analyzed by 2-tailed Student’s *t* test (for comparisons of 2 groups) and ANOVA (for comparisons of 3 or more groups), where appropriate. Data for survival were analyzed by Log-Rank (Mantel-Cox) Test. Two-sided *p* < 0.05 was considered statistically significant (*P* < 0.05 (^*^), *P* < 0.01 (^**^), *P* < 0.001 (^***^), *P* < 0.0001 (^****^)).

## SUPPLEMENTARY MATERIALS FIGURES


