# Supplementary material for: Lysis-independent potentiation of immune checkpoint blockade by oncolytic virus
Source: Oncotarget. 2018 Jun 19;9(47):28702–16. doi: 10.18632/oncotarget.25614 (PMC6033351; doi:10.18632/oncotarget.25614)
Supplement: Supplementary file 1 [file oncotarget-09-28702-s001.pdf]

Lysis-independent potentiation of immune checkpoint blockade by oncolytic virus

SUPPLEMENTARY MATERIALS

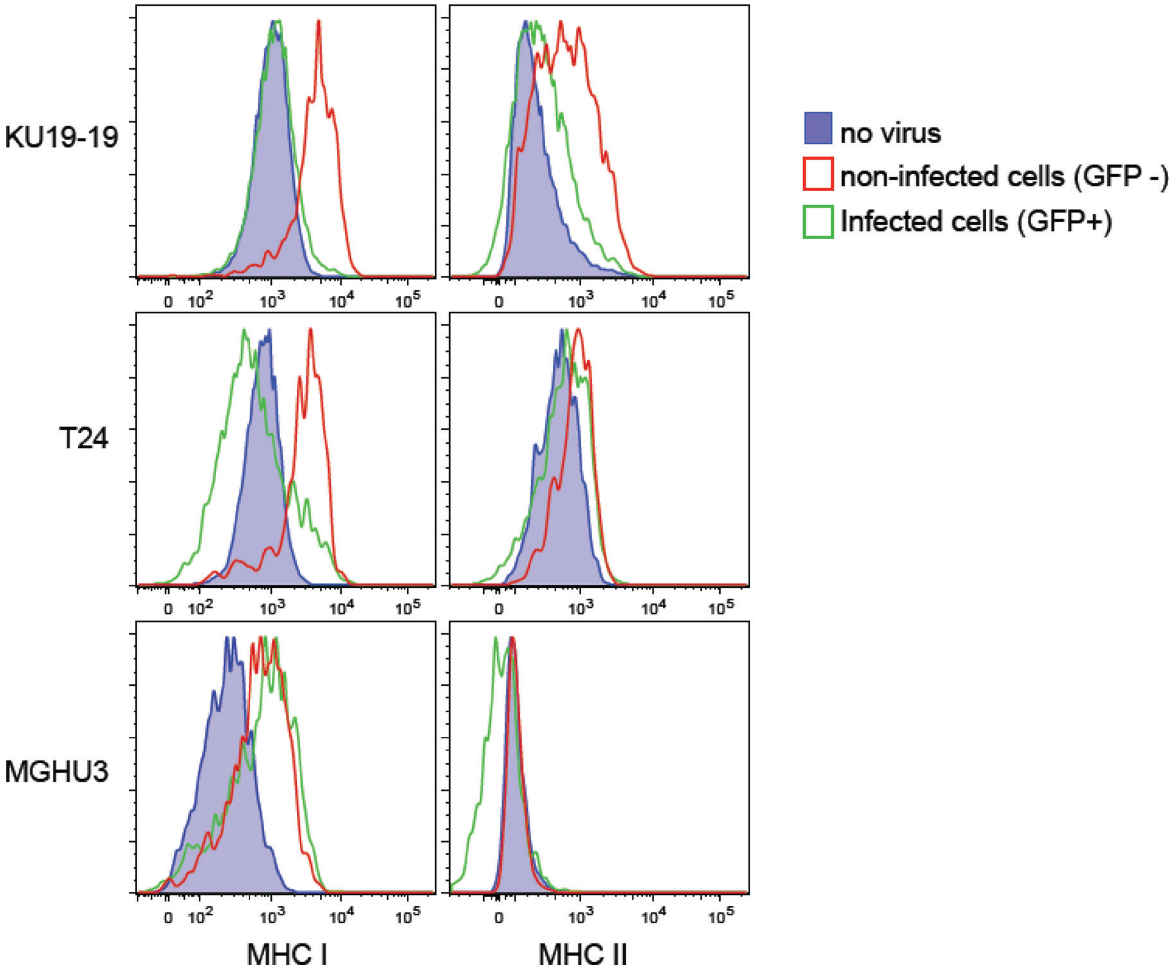

Supplementary Figure 1: NDV infection upregulates MHC I and MHC II in the infected and non-infected cells. Indicated cell lines were treated with NDV expressing GFP (NDV-GFP) at MOI of 2 and 24 hours were collected by scraping, stained with antibodies to HLA-ABC (MHC-I) and HLA-DR (MHC-II), and analyzed by flow cytometry. Representative histograms represent gating on the NDV-infected (GFP+), non-infected (GFP−), and control cells that received no virus.

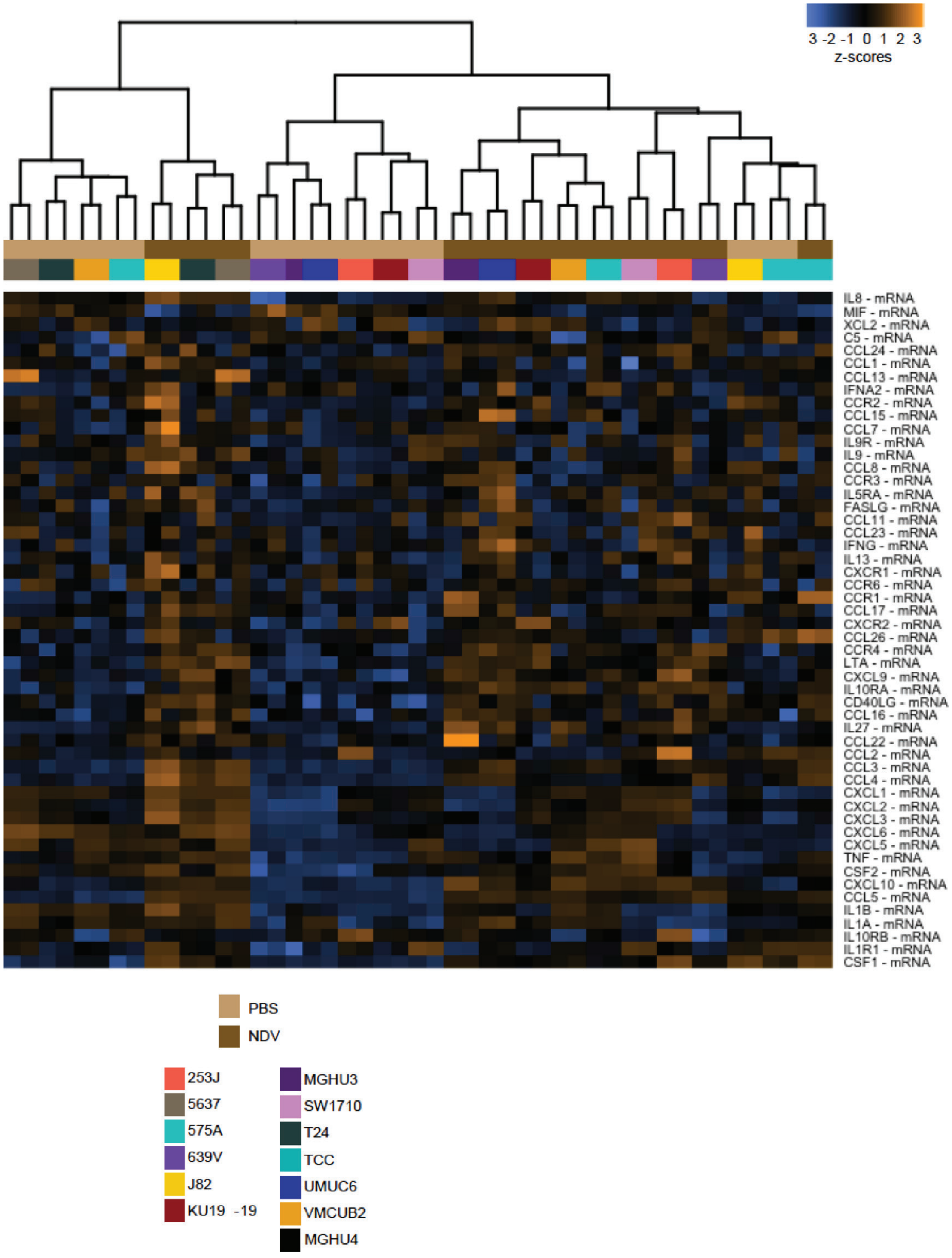

Supplementary Figure 2: NDV induces upregulation of chemokines and cytokines in human bladder cancer cell lines. Gene expression analysis was performed using the Innate Immunity Panel profiling kit on the Nanostring platform. Upregulation of gene sets related to chemokine and cytokine response is shown. Data represent a single experiment with 2 replicates per cell line.
